# Hallucinations at the interface of philosophy and the empirical sciences

**DOI:** 10.3389/fpsyg.2025.1699432

**Published:** 2025-11-25

**Authors:** Nevia Dolcini

**Affiliations:** 1 Department of Philosophy and Religious Studies, University of Macau, Macao SAR, China; 2 Centre for Cognitive and Brain Sciences, Institute of Collaborative Innovation, University of Macau, Macao SAR, China

**Keywords:** bottom-up models, hallucination, insight, mental imagery, perception, predictive processing, top-down models

## Abstract

Hallucinations sit at the crossroads of philosophy and the empirical sciences, but are often approached with divergent aims. In philosophy, they are mainly treated stipulatively as experiences subjectively indistinguishable from veridical perception and used to probe theories of perception, justification, and consciousness. Empirical research, by contrast, investigates heterogeneous, clinically embedded hallucinatory phenomena, many of which differ phenomenologically from ordinary perception. This paper diagnoses the conceptual misalignment that follows from this divergence and offers a preliminary framework to narrow it through conceptual analysis. Rather than advancing a single unifying theory, I clarify key distinctions, including indistinguishability, insight, sense of reality, agency, and ownership, and sketch points of contact with constructs in the sciences. First, I examine leading empirical models—bottom-up, top-down, and predictive processing—highlighting what each explains and where each is limited. Second, I re-situate hallucinations within core philosophical debates on perception, mental imagery, and phenomenology, showing how empirical findings both inform and complicate current accounts. Third, I assess interdisciplinary developments that challenge unitary models and support pluralist, integrative approaches. Hallucinations are thus recast as a family of related phenomena, and the analysis provides theoretical coordinates for more productive interaction between philosophy and science.

## 1 Introduction

Hallucinations have long occupied a central place in both philosophy and the empirical sciences, yet the two domains approach them in strikingly different ways. In philosophy, they are typically defined *ex hypothesi* as experiences subjectively indistinguishable from veridical perception yet occurring in the absence of any corresponding external stimulus. So construed, hallucinations function less as empirical phenomena to be measured than as theoretical tools to challenge core assumptions about perceptual justification, the relation between mind and world, and the nature of consciousness (Grice, 1961; Martin, 2004; Crane, 2006). In particular, hallucinations have served as focal points in two major debates. The first, in epistemology, asks whether perception alone can justify empirical beliefs—an issue linked to disputes over internalism vs. externalism and theories of justification such as foundationalism and coherentism (BonJour, 1985; McDowell, 1994; BonJour and Sosa, 2003). The second, in metaphysics of perception, asks whether perception is best understood as a direct relation to external objects or as mediated by representational states—an issue at the heart of debates between direct realism, representationalism, and sense-data theories (Snowdon, 1992; Campbell, 2002; Fish, 2009). Crucially, these debates rely on the stipulative notion of hallucination and make no appeal to actual cases.

By contrast, psychology, psychiatry, and neuroscience study hallucinations as concrete and often pathological phenomena, since they are frequently associated with psychiatric conditions such as schizophrenia, delusional disorder, and dementia, as well as with neurological conditions including Parkinson’s disease and Charles Bonnet syndrome (Larøi et al., 2012; Linszen et al., 2022). However, hallucinatory experiences extend beyond clinical conditions: they are reported in the general population, particularly in hypnagogic and hypnopompic states or under the influence of psychoactive substances (Iudici et al., 2019). In this context, the term “hallucination” is used descriptively to denote observable phenomena reported by subjects: empirical models seek to explain their mechanisms, whether through bottom-up sensory disturbance accounts, top-down cognitive dysfunction models, or integrative frameworks that combine both, such as predictive processing theories (Frith, 1992; Hugdahl, 2009; Powers et al., 2016; Vance and Stokes, 2017).

This divergence yields a conceptual misalignment. Philosophical treatments of hallucinations assume their indistinguishability from veridical perception—a premise that grounds debates between representationalist and disjunctivist theories of perception (Siegel, 2006; Farkas, 2013a; Macpherson, 2013; Moran, 2024). Empirical findings, however, show that many hallucinations differ phenomenologically from ordinary perception: they may be fragmentary, inconsistent, or accompanied by a diminished sense of reality (ffytche, 2013). Moreover, in many cases, subjects retain ‘insight’ into the hallucinatory character of their experiences, distinguishing them from genuine perceptions. This is often taken to mark the distance between ‘perfect’ philosophical hallucinations and actual cases (Farkas, 2013b). From this angle, philosophy and science can seem to address different objects: one abstract and hypothetical (i.e., a conceived possibility); the other concrete and heterogeneous. The former serves to stage epistemological and metaphysical disputes, while the latter designates phenomena to be explained in clinical and cognitive terms. This alleged gap has led some philosophers to question whether purely hypothetical cases should remain central to debates on perception, and to argue that attending to actual hallucinatory phenomena may more effectively enhance our understanding of perception, imagery, and experience (Dokic and Martin, 2015; Ratcliffe, 2017). Empirical researchers, for their part, have called for interdisciplinarity, maintaining that progress on hallucinations requires sustained collaboration with philosophy (Wilkinson et al., 2022).^1^ Despite repeated calls for interdisciplinarity from both sides, however, what is often counted as fruitful is philosophical work strictly aligned with empirical concerns, while the conceptual core that has historically defined hallucinations in philosophy, namely, indistinguishability, is regarded more as a theoretical construct than an essential component of ‘real’ hallucinations. This asymmetry narrows the terms of engagement, thereby potentially constraining prospects for genuinely two-way collaboration.

Granted that the concept of hallucination is neither exhausted by mere logical possibilities nor reducible to anomalous experiences, this paper aims to foster an interdisciplinary approach to the study of hallucinations by forging a closer alignment between the conceptual frameworks of philosophy and empirical research. I pursue this alignment through conceptual analysis, clarifying core distinctions in the scientific and philosophical literature and analyzing how hallucinations are theorized across domains.

The paper proceeds as follows. Section 2 surveys leading empirical models of hallucination—distinguishing between bottom-up, top-down, and predictive processing approaches—and their theoretical commitments. Section 3 situates hallucinations within key philosophical debates on perception, mental imagery, and the phenomenology of experience, showing how empirical findings complicate or destabilize purely hypothetical treatments. Section 4 turns to recent interdisciplinary developments that challenge the assumption of a unitary model of hallucination, suggesting more pluralistic and integrative directions, and it concludes with implications for future work across philosophy and the sciences.

## 2 Empirical models of hallucination

Empirical research has sought to explain hallucinations in terms of perceptual and cognitive mechanisms, developing models that reflect broader assumptions about the nature of perception. In this context, hallucinations are typically defined as perceptual-like experiences occurring in the absence of corresponding sensory stimulation from the external environment (David, 2004). They can take a variety of forms and may involve one or more sensory modalities; some are monomodal (e.g., auditory hallucinations), while others are multimodal, engaging several or even all sensory channels (Pienkos et al., 2019; Toh et al., 2021). Their nature, frequency, and underlying mechanisms vary considerably across clinical, neurological, and psychological conditions, which makes hallucinations a complex and multifaceted object of study. Clinically, hallucinations are most often associated with psychotic disorders—conditions characterized by a loss of contact with reality, including schizophrenia, schizoaffective disorder, and substance-induced psychosis (Waters and Fernyhough, 2017). For example, auditory hallucinations are most strongly linked to schizophrenia, while visual hallucinations occur more frequently in dementia, delirium, and substance-induced states (Collerton et al., 2012). Because research in this area is primarily motivated by the need for therapies targeting psychotic symptoms, most studies to date have focused on clinical populations. As a result, auditory hallucinations and visual hallucinations—the most common in both clinical and non-clinical contexts (Linszen et al., 2022)—remain the most extensively studied.

Notably, however, the taxonomy of abnormal experiences remains a matter of debate. Consider, for instance, the traditional distinction between illusions and hallucinations: both depart from veridical perception, yet they do so in different ways. Illusions are usually thought to involve a genuine perceptual component—the object is present but misperceived, appearing other than it is (Smith, 2002). Hallucinations, by contrast, are typically regarded as *non-perceptual* in nature, insofar as they lack any external object to which the experience can be traced. This might suggest that hallucinations and illusions are fundamentally distinct (Liu et al., 2019). Still, the boundary between them is more blurred than commonly assumed. Philosophical analyses^2^ and neurobiological findings^3^ alike indicate that the two phenomena share underlying features—a view already anticipated by phenomenologists such as Husserl and Merleau-Ponty, who regarded both as forms of perceptual error (Overgaard, 2022). Clinically, too, the distinction often breaks down: patients who at times ‘see things that are not there’ may at others ‘see real things incorrectly’. Several factors contribute to the diagnostic challenge of sharply differentiating between illusions and hallucinations. On the one hand, certain perceptual anomalies—such as polyopia (‘seeing’ multiple images of a single object) and metamorphopsia (e.g., faces appearing deformed)—can accompany both veridical and hallucinatory experiences, suggesting a partial overlap in underlying mechanisms (ffytche, 2004). On the other hand, some *prima facie* illusory experiences, like paraedolia, where meaningful forms like faces or animals spontaneously emerge from unstructured stimuli (e.g., curtains, walls, or clouds), are phenomenologically close to hallucinations (Uchiyama et al., 2012). Moreover, recent studies indicate that hallucinations can be triggered by specific elements of the visual environment (Collerton et al., 2024), further complicating any strict theoretical or clinical separation between internally generated and externally modulated experiences. These difficulties suggest that what counts as a ‘real’ hallucination is far from self-evident, as the term may cover a spectrum of experiences with varied phenomenology.

Empirical research has approached this heterogeneity through different accounts, each emphasizing distinct aspects of perceptual and cognitive processing. Three families of explanatory models of hallucinations have been particularly influential: *bottom-up accounts*, which regard them as stemming from a malfunction of the sensory pathway; *top-down accounts*, which stress cognitive dysfunction and misattribution; and *predictive processing approaches*, which integrate both perspectives within a Bayesian framework. Each highlights different aspects of perceptual and cognitive processing, and their contrasts are a clear mark of the conceptual and empirical complexity of hallucinatory phenomena. Reviewing these main empirical models is necessary not only to map the scientific terrain but also to clarify the conceptual assumptions that connect each of them with major positions in the philosophical debate on hallucination. Bottom-up approaches exhibit affinities with empiricist traditions and many forms of representationalism, which explain anomalous experiences primarily in terms of sensory-level disturbance or misrepresentation. By contrast, top-down accounts align with cognitivist, inferential, and constructivist views that emphasize theory-ladenness, prior expectations, and metacognition in shaping experience. Integrative frameworks, such as predicting processing, parallel hybrid accounts that seek to situate cognitive, affective, and perceptual factors within a unified architecture. Hence, the three empirical families surveyed below also prepare the ground for the philosophical analyses developed in Section 3.

### 2.1 Bottom-up models

Bottom-up models explain hallucinations as disruptions in perceptual systems. They locate the source in atypical activity of early sensory areas (e.g., V1 in vision), where mechanisms that normally support veridical perception become spontaneously active in the absence of external stimulation. The internally generated ‘noise’ is then processed as if it were genuine input, and under certain conditions, autonomous neural activity can generate hallucinatory experience (Ermentrout and Cowan, 1979; Bressloff et al., 2002). A key observation is that many simple hallucinations—whether induced experimentally (e.g., flicker or magnetic fields), triggered by drugs such as mescaline, or arising in clinical conditions like migraine and epilepsy—exhibit recurring geometric patterns known as “Klüver forms” (Klüver, 1926, 1966). These are thought to reflect the functional organization of the visual cortex and exemplify self-organized activity within excitatory-inhibitory neural networks (Billock and Tsou, 2012). More complex images are thought to arise when activity spreads from V1 to higher visual areas, which integrate and feed back on the initial signal to produce increasingly elaborate hallucinatory content. Although some studies show that the amplitude of V1 activity is smaller than that observed in downstream regions (ffytche, 2008; Allen et al., 2008), the evidence remains consistent with the view that hallucinations are triggered in V1 and then propagate to higher areas.

Converging findings support a close correspondence between neural activity in veridical perception and hallucination. Pearson et al. (2016) showed that both induced hallucinations (via flicker stimulation) and the veridical perception of a gray blob arise in the visual cortex. Similarly, EEG studies found that the same flicker frequencies, which preferentially induced radial or spiral hallucinations, also enhanced oscillatory responses when participants viewed corresponding static images (Mauro et al., 2015). Further evidence for the role of lower-level perceptual processing in hallucinations comes from post-lesion studies. These show that damage is almost always localized to the sensory pathway of the affected modality and is often accompanied by compensatory hyperactivation in adjacent tissue (Braun et al., 2003). Such findings suggest that intact sensory circuits normally dampen the intensity of internal representations; when this inhibitory function is lost, hallucinatory activity is ‘released.’^4^

A well-known example comes from the patterned hallucinations observed in Charles Bonnet syndrome, where visual loss correlates with extremely vivid yet bizarre visual hallucinations (Menon et al., 2003; Schadlu et al., 2009). These cases gave rise to the “deafferentation theory,” according to which a decrease in sensory input (i.e., deafferentation) lowers thresholds for neural activation, resulting in hyperexcitability of cortical circuits and the spontaneous generation of hallucinatory content (Burke, 2002; ffytche, 2007b; Marschall et al., 2020). Related evidence shows that impairments in lower-level visual processing and attention are more pronounced in Parkinson’s patients who experience visual hallucinations (Weil et al., 2016), and that hearing impairment or lesions in auditory pathways are associated with psychosis onset (Thewissen et al., 2005).

Bottom-up models are thus attractive for their ability to link neural pathology directly to phenomenology. Yet they are limited in scope: while they account well for simple (visual) hallucinations, they struggle to explain complex, content-rich, or thematically organized experiences, where higher-level constraints (memory, affect, expectation, attention) and multimodal integration seem indispensable.^5^

### 2.2 Top-down models

Top-down models approach hallucinations as the outcome of *cognitive* rather than *perceptual* dysfunction. Whereas bottom-up accounts were largely developed in research on induced hallucinations and neurological disorders, top-down approaches arose primarily within the study of psychosis. Unsurprisingly, they are often tailored to explain auditory verbal hallucinations (AVH), a hallmark symptom of schizophrenia and other psychotic disorders (Tarrier, 2008; Larøi et al., 2012).

The central assumption of these approaches is that hallucinations originate in otherwise *ordinary* cognitive states which, due to failures at higher levels of processing, are misinterpreted as perceptual. Such states—commonly inner speech or mental imagery—are not abnormal in themselves, but become hallucinatory when, for example, attributed to an external source. Importantly, even if not intrinsically pathological, these states may still be experienced as highly distressing once misinterpreted or recognized as self-generated. Some have argued that the distress associated with hallucinations, particularly in schizophrenia, stems less from the voices themselves than from how they are interpreted or processed at a metacognitive level (Morrison, 1998; Larøi and Van der Linden, 2005). In turn, metacognitive appraisal may also generate distress (i.e., anxiety and panic) potentially triggering hallucinatory experiences. Indeed, metacognitive beliefs about the need for cognitive consistency have been shown to amplify distress in individuals experiencing auditory hallucinations, suggesting a moderating role in the appraisal–distress relationship (Choudhary et al., 2022).

Consequently, top-down models must account for two aspects–though they differ in how they do so: (i) the cognitive state that underlies the experience, and (ii) the mechanism that explains why such a state ends up being experienced as hallucinatory. *Inner speech* and *imagery* are the most common candidates for (i), while failures in metacognitive processes, particularly in reality and source monitoring, are typically invoked to account for (ii). Although psychiatrists had long described hallucinations in terms of a disruption of inner–outer boundaries, Hoffman (1986) was among the first to propose a cognitive-processing model of AVHs. He regarded them as *ordinary verbal images* accompanied by an unusual sense of ‘unintendedness’, arising from a disruption in language-planning processes (Posey, 1986). When such images are incoherent with the subject’s cognitive goals, the resulting sense of unintendedness may lead to misattribution—that is, the experience is ascribed to an external source. While aspects of Hoffman’s model have been criticized,^6^ the misattribution framework has remained central. Several of his insights have been further explored, such as the role of incoherence in AVHs (Larøi and Van der Linden, 2005). It has been suggested that misattribution may arise from inconsistencies between intrusive thoughts and metacognitive beliefs, sustained by efforts to reduce cognitive dissonance (Morrison et al., 1995; Morrison et al., 2000). Building on this framework, Bentall (1990) argued that misattribution can also arise from a more general deficit or bias in the inferential processes required to discriminate between *real* (i.e., perceived) and *imaginary* (i.e., self-generated) events. The association between dysfunctions in this mechanism—known as self- or source-monitoring—and schizophrenia has been extensively studied (Bentall et al., 1991; Frith et al., 1991) and repeatedly linked to hallucinations (see also Beck and Rector, 2005).

Building on this, the most widely accepted account explains misattribution in terms of inner speech rather than verbal imagery more broadly (Frith, 1992, 2015). The idea, which traces back to Viktor Kandinsky, is supported by evidence of subvocalization (Gould, 1949; Green and Preston, 1981) and speech musculature activity (McGuigan, 1966) during AVHs, mirroring what is observed in ordinary inner speech and thought. Consistently, the engagement of speech muscles that normally block subvocalization tend to inhibit the AVHs (Bick and Kinsbourne, 1987). Furthermore, research on source monitoring suggests that impairments in distinguishing between self-generated and externally generated speech play a crucial role in AVHs. Fernyhough (2004) further refined this account by suggesting that they arise from transitions between expanded and condensed forms of inner speech. On this view, ordinary cognitive activity can become experienced as alien when its phenomenological features—such as fragmentation, compression, or lack of agency—conflict with the subject’s expectations about self-generated thought.

The scope of top-down models has also been extended beyond the auditory domain. Hallucinations in other modalities have been linked to disruptions in mental imagery more broadly, where failures to maintain cognitive control over imagery result in its intrusion into perceptual processing (Nanay, 2016). Nonetheless, despite their explanatory range, purely top-down models face difficulties: they account well for the cognitive misattribution component, but less so for the vivid sensory qualities of many hallucinations and their close ties to early perceptual mechanisms.

### 2.3 Predictive processing models

Predictive processing models aim to dissolve or reconceptualize the traditional divide between bottom-up and top-down accounts.^7^ Rooted in Bayesian brain theory, they view perception as an active, generative process of hypothesis testing: the brain continuously generates predictions about incoming sensory input and updates them in light of prediction error (Corlett, 2019). Hallucinations arise when this inferential balance is disrupted, such that top-down priors are assigned excessive weight relative to sensory evidence, leading to perceptual inferences detached from reality (Powers et al., 2016).

These models reframe hallucinations as a product of misweighted inference in hierarchical coding systems, rather than mere sensory noise or isolated cognitive errors. For example, Waters et al. (2012) propose that AVHs involve both aberrant low-level sensory signals (a bottom-up contribution) and dysfunctional high-level influences such as attention, prior experience (memory), or affective states (a top-down contribution). Although they identify perceptual activity at the origin of auditory visual hallucinations, the form and content of the hallucinatory experience are ultimately determined by top-down mechanisms, which remain continuous with those shaping ordinary perception. At the same time, since these top-down mechanisms, although dysfunctional in the case of hallucinations, are the same as those involved in typical perception, the experience cannot be said to be fundamentally different in kind from perception. Even bottom-up accounts such as deafferentation have been reformulated in predictive terms: here, lesions or sensory impairments reduce the informativeness of bottom-up input, causing the system to rely disproportionately on internally generated predictions, which can then manifest as hallucinations (O’Callaghan et al., 2017).

Several predictive processing models suggest that under conditions of cognitive strain, the balance between higher- and lower-level inferences may be disrupted, shifting the system toward faster, less accurate predictions; in stressful situations, this costly process may be abandoned in favor of faster—but impaired—predictions, resulting in hallucinatory experiences. Neuroimaging evidence lends further support to predictive processing models. In an fMRI study of patients with schizophrenia who experienced daily AVHs, Horga et al. (2014) found that hallucinatory episodes were associated with reduced prediction-error signals in the auditory cortex. Further work reinforces the predictive coding framework, presenting it as a transdiagnostic account of cognitive dysfunction. A PRISMA-guided review of 72 studies found heterogeneous impairments across disorders: schizophrenia was consistently associated with deficits in non-social predictive processes, while autism showed selective impairments in social prediction (Qela et al., 2025). At the same time, computational psychiatry reviews caution against overly canonical and monolithic formulations of predictive coding, noting that anomalies differ between hallucinations and delusions, that priors may be abnormally strong or weak depending on context, and that disruptions may vary across sensory modalities or hierarchical levels (Sterzer et al., 2018).

Taken together, these findings suggest that predictive coding provides a powerful unifying framework for understanding hallucinations, while also revealing important limitations. On the one hand, it elegantly explains hallucinations as exaggerations of normal inferential mechanisms arising from misweighted interactions between priors and sensory evidence. On the other hand, the heterogeneity observed across modalities, clinical populations, and hierarchical levels indicates that no single predictive deficit can explain all hallucinatory phenomena. Thus, rather than offering a uniform account, predictive models are best seen as mapping a spectrum of possible disruptions within the same inferential architecture. This perspective preserves the continuity between hallucination and ordinary perception—both drawing on the same mechanisms, albeit misfiring under atypical conditions—while acknowledging variability that resists one-size-fits-all explanations. In this respect, predictive processing is especially promising as a framework for bridging empirical and philosophical approaches, but it must be developed in ways that accommodate pluralism rather than strict unification.

### 2.4 Heterogeneity and the limits of unitary models

Although these models provide powerful explanatory tools, they also highlight the heterogeneity of hallucinatory phenomena. Hallucinations associated with sensory deprivation, neurological lesions, psychotic disorders, and sleep states may differ not only in their phenomenology but also in their neurobiological underpinnings (ffytche, 2013; Linszen et al., 2022). Attempts to fit all forms into a single explanatory framework risk obscuring meaningful distinctions. For this reason, some researchers argue for abandoning the idea of a unitary category of “hallucination” in favor of more fine-grained taxonomies (Ratcliffe, 2017; Wilkinson and Ratcliffe, 2017). This perspective dovetails with phenomenological approaches in philosophy, which emphasize differences in sense of reality, ownership, and affective tone across hallucinatory experiences.

## 3 Philosophical accounts of hallucination

Philosophical debates on hallucination have long shaped theories of perception, consciousness, and mental representation. Since at least Grice’s (1961) Causal Theory of Perception, hallucinations have been regarded as a crucial test case. The guiding idea is that any serious theory of perception must explain how an experiential state can arise that feels subjectively identical to a perceptual one, even in the absence of an external object. A satisfactory account must therefore do two things: explain why hallucinations and veridical perceptions can appear indistinguishable to the subject, and at the same time mark the distinction between genuine (perceptual) and non-genuine (hallucinatory) cases. From this perspective, hallucinations function less as empirical phenomena to be measured than as conceptual tools for probing the limits of perception and its epistemic role. In what follows, I examine three domains where this role has been especially prominent: (i) theories of perception, (ii) accounts of mental imagery, and (iii) analyses of the phenomenological features of experience.

### 3.1 Hallucinations and theories of perception

The most prominent role of hallucinations in philosophy lies in debates about the nature of perceptual experience. The central question is whether the phenomenal and metaphysical structures of hallucinations and veridical perceptions are of the same fundamental type.

Representationalists, or common kind theorists,^8^ argue that hallucinations and veridical perceptions are of the same type, differing only in their causal origins. They often regard perception as mediated by a representational state, so that experiences have correctness or veridicality conditions: an experience as of a black horse may represent the world as being different from the way it is–perhaps radically so, as in the case that there is no black horse (Dretske, 1995; Tye, 2007). Thus, the difference between a hallucinatory experience of seeing a black horse and the corresponding veridical perception is not intrinsic to the experience itself. Rather, it is determined by factors such as the etiology of the experience, its relations to other experiences, or even its integration with the subject’s beliefs. Since both hallucinations and perceptions can involve the very same representational state, they are subjectively indistinguishable. This supports a unified ontology of perceptual experience.

By contrast, naïve realism or relationalist theories deny that hallucinations and perceptions are states of the same kind. Perception is not mediated by representational content but consists in a relation of acquaintance with the mind-independent world. For example, a veridical perception of a black horse is a *relation* of ‘acquaintance’ with the horse itself (the mind-independent object) and/or some of its properties (Campbell, 2002; Fish, 2009). In hallucination, no such relation exists (i.e., there is no object one is acquainted with); hence, the subject cannot be in the same experiential state. This motivates disjunctivism, which holds that hallucinations and perceptions are fundamentally different, sharing only the property of being indiscriminable from the subject’s perspective (Crane and French, 2016). The debate thus turns on the role of *indistinguishability*: representationalists take it as evidence of a common experiential kind, while disjunctivists treat it as an epistemic fact with no ontological implications. The latter position has been further developed in the negative epistemic account (Martin, 2004), holding that nothing substantive can be said about hallucinations beyond their indiscriminability from veridical perception.

Some representationalist theories, grounded in causal accounts of perception, attempt to bridge this gap by treating hallucinations as representational states triggered internally rather than by external stimuli. Employing such a proximate cause-same effect can explain why hallucinations and perception are *experientially the same*. Disjunctivists, however, deny that such a strategy is viable, since in their view, hallucinations cannot be assimilated to perceptual states at all.^9^ One interesting problem about this debate or alleged chasm, as noted by Crane (2006), is whether these philosophical accounts genuinely engage with actual hallucinations as studied in empirical science or instead rely on highly idealized, ‘philosophical’ hallucinations.

Recent work has sought to move beyond purely hypothetical examples and to test philosophical accounts against real cases. Allen (2015) offers an explicit example of this methodological stance, proposing that theories of hallucination should be informed by the empirical diversity of *actual* hallucinatory phenomena rather than by the highly idealized ‘philosophical’ case. Along these lines, Coates (2013) examines musical hallucinations to probe theories of transparency, while Milkowski (2017) argues that representationalist frameworks are particularly well-suited to computational models of hallucination. Dokic and Martin (2015) discuss various hallucinatory cases to show that perception and the sense of reality can come apart: actual hallucinations may involve a vivid feeling of presence, whereas genuine perceptions may lack it, as in derealization. This dissociability, they argue, undermines the idea of a single experiential kind common to both (i.e., subjective indistinguishability does not entail experiential identity), thereby lending support to disjunctivism.

Yet these attempts also expose a central tension: even when philosophers turn to empirical cases (e.g., Patel, 2025), it remains unclear whether the same notion of ‘hallucination’ is operative across philosophical and empirical domains. This tension may in part reflect differences in explanatory aims both across and, to some extent, within domains.^10^ While such differences may lead to equivocation about the explanandum, they may also prove epistemically fruitful, as conceptual analysis and empirical investigation refine each other’s understanding of what counts as a hallucinatory experience (i.e., the explanandum itself). One might object that philosophy and science genuinely address different explananda. For instance, it may be argued that perception science theorizes about psychological kinds (subpersonal and non-factive states that underlie perceptual processing), whereas philosophy—as in the naïve realist tradition—investigates ordinary kinds of perceptual experience, conceived as conscious and factive relations to the world (French and Phillips, 2023). In this sense, philosophy and science may seem to target different explananda: the former concerned with the experiential or phenomenological character of perception, the latter with its subpersonal mechanisms. Yet, as Siegel (2007) observes, the structure and content of perceptual experience can be examined through both conceptual and empirical means. Her method of phenomenal contrast exemplifies how phenomenological description and experimental evidence can converge on the same explanandum (i.e., the character and representational content of experience) while preserving distinct explanatory roles. Finally, from a more explicitly integrative perspective, Khalifa et al. (2022) offer a general epistemological framework that helps to articulate this kind of interdisciplinary convergence. Their model of ‘understanding-based integration’ explains how distinct explanatory approaches can be systematically related insofar as they collectively promote understanding. What makes this view particularly relevant here is that it conceptualizes *explanatory pluralism* not as fragmentation but as a mark of epistemic progress. On this account, multiple explanations can jointly enhance understanding by identifying inter-explanatory relations—constraints, complementarities, or hierarchical dependencies—that enrich our grasp of the phenomenon. In this sense, philosophical and empirical explanations can be seen as coordinated contributions to a shared epistemic enterprise, differing not in their object of inquiry but in the methods and inferential strategies through which they access and articulate it.

### 3.2 Hallucinations and mental imagery

A second line of philosophical debate situates hallucinations within broader accounts of mental imagery. Traditionally, hallucinations have been contrasted with imagery by their involuntariness, vividness, and perceptual character. Yet this distinction is contested. For example, it has been argued that hallucinations are best understood as degenerate kinds of sensory imagination rather than degenerate kinds of perceptual experience (Allen, 2015), or as forms of mental imagery that have escaped cognitive control (Nanay, 2016). On this view, the difference is not one of kind but of degree: hallucinations are images that acquire the immediacy and salience of perception. Thomas (2014), for instance, challenges McGinn’s (2004) sharp distinction between imagination and perception, suggesting that empirical research on hallucinations undermines the assumption that imagery is always under voluntary control; thus this classification is unfounded. If so, imagery cannot be regarded as entirely voluntary, but at times takes on the involuntary and compelling character typical of perception. This brings imagery and perception closer together than McGinn’s strict dichotomy allows.

The influence runs in both directions: one’s conception of hallucination informs theories of mental imagery, and theories of imagery shape how hallucinations are understood. As Nanay (2016) emphasizes, even if hallucinations are classified as imagery rather than perception—as disjunctivists propose—this need not imply a sharp separation from perception. If mental imagery itself shares essential features with perception, then hallucinations, while a form of imagery, cannot be considered radically different from perceptual states. Conversely, if imagery is treated as categorically distinct from perception, hallucinations must be regarded as *sui generis* phenomena. Empirical findings complicate this philosophical terrain. Evidence that inner speech and visual imagery underlie certain hallucinations (see § 2.2) suggests continuity rather than categorical division. Yet the precise boundary between imagery, perception, and hallucination remains unsettled, underscoring the conceptual instability of the category itself.

### 3.3 Hallucinations and features of experience

Beyond theories of perception and imagery, hallucinations have been employed to investigate specific features of conscious experience, including sense of agency, ownership, and reality. AVHs have played a particularly relevant role in debates on the experience of agency: Wegner (2002, 2003, 2004) interprets them as evidence that the sense of control or authorship that normally accompanies thought and experience is an *illusion*, generated when mental events satisfy certain ‘consistency conditions.’ On his account, we attribute authorship to our thoughts when they cohere with prior states; hallucinatory voices, by contrast, reveal how this feeling of control can be disrupted. Drawing on Hoffman’s cognitive model for AVHs (see §2.2), Wegner argues that inconsistency with prior thoughts leads subjects to attribute voices to an external source, while consistency underlies the ordinary experience accompanied by a sense of control and of deliberation.^11^ Subsequent work has challenged this interpretation. Meynen (2010) objects that reliance on inconsistency is misplaced: incoherence is indeed a cognitive trait commonly displayed in schizophrenia, but it is neither the prevailing explanation of AVHs nor a necessary condition for the ‘suspension’ of agency.

A related line of inquiry concerns the *sense of ownership*. Maiese (2018) contends that a suspension of ownership may be the unifying feature of the otherwise phenomenologically diverse class of AVHs. Based on evidence from schizophrenia, she links this to failures of selective attention, which is central to executive functioning and impaired in many patients. On this view, AVHs provide a window onto how ownership depends on attentional mechanisms and their integration within agency. A further dimension concerns the *sense of reality* that accompanies hallucinatory experience, that is, the sense that the sensory content is real. Certain types, including psychedelic visions and Charles Bonnet syndrome hallucinations, are often marked by a diminished sense of reality, with subjects typically retaining insight into their non-veridical character. By contrast, AVHs in psychosis are more frequently experienced as fully real. Philosophers have debated whether such a ‘sense of reality’ is intrinsic to perception or cognitively modulated (Siegel, 2006; Farkas, 2013b; Fortier, 2018). Interestingly, cross-cultural research shows that concepts of reality embedded in cultural traditions shape both the threshold for hallucinations and the degree to which they are experienced as real, with attitudes toward hallucinations influencing emotional response and perceived controllability (Al-Issa, 1995).

Finally, hallucinations intersect with discussions of the minimal self. Work in phenomenological psychopathology (Sass and Parnas, 2003; Zahavi, 2005) interprets hallucinations as disruptions of basic self-experience, including ownership and first-person perspective. This line of research has been especially influential in bridging philosophy, psychiatry, and clinical phenomenology, showing how anomalous experiences can provide relevant information about the structural features of subjectivity.

## 4 Interdisciplinary perspectives: desiderata, challenges, and implications

Recent years have seen the growth of interdisciplinary approaches that integrate phenomenological insights, clinical psychiatry, and cognitive neuroscience to move beyond reductive or overly abstract accounts of hallucinations. I highlight three themes where such dialogue is especially productive: pluralism, the cognition vs. perception distinction, and ‘insight’ as a bridging dimension. I then draw the implications and challenges for a shared research program.

Phenomenological psychopathology has long emphasized that hallucinations cannot be understood solely in terms of stimulus absence or misrepresentation. It has been argued that hallucinations reveal disruptions in the ‘minimal self’—the basic sense of ownership and first-person perspective that ordinarily structures experience (Sass and Parnas, 2003; Zahavi, 2005). On this view, AVHs in schizophrenia is not regarded as a mere misattributions of inner speech but reflect deeper disturbances of subjectivity. This perspective underscores the *heterogeneity* of hallucinatory phenomena: experiences that appear *prima facie* similar may differ significantly in their experiential structure, while apparently different experiences may overlap. For example, the phenomenology of ‘hearing voices’ in schizophrenia can intersect with thought insertion, blurring standard diagnostic boundaries (Ratcliffe, 2017; Wilkinson and Ratcliffe, 2017). Clinical psychiatry has likewise moved away from treating hallucination as a single category of phenomena. ffytche (2007b) and ffytche et al. (2010), among others, distinguish between classes of hallucinations—those linked to psychosis, sensory deprivation, or neurodegenerative conditions—on both phenomenological and neurobiological grounds. On this view, visual hallucinations in Parkinson’s disease, the complex imagery of Charles Bonnet syndrome, and AVHs in schizophrenia all fall under the same label but differ substantially in mechanism and experiential profile. A pluralistic orientation, therefore, seems warranted: hallucinations are better mapped as subtypes correlated with neural processes and lived structures rather than collapsed into a homogeneous kind.

Interdisciplinary research has the potential to enhance our understanding of how cognitive and perceptual levels of processes interlock by mapping subtypes of hallucinatory experience and by correlating first-person profiles with third-person quantitative and behavioral measures. Such mapping not only may improve diagnostic precision but also enrich philosophical accounts of perception and cognition. Neurophenomenology offers one promising path by combining first-person reports with neural data (Varela, 1996; Gallagher and Zahavi, 2008). In the context of hallucinations, this means complementing cognitive and neuroscientific models with fine-grained accounts of how different senses of reality, agency, or ownership manifest in lived experience. Predictive coding theories, for instance, gain depth when supplemented with phenomenological descriptions of variability in the felt ‘presence’ or ‘sense of reality’ of the hallucinatory content (Corlett, 2019). Integrative models need not erase theoretical differences. Bottom-up accounts illuminate pattern-like or lesion-related phenomena; top-down models clarify misattribution and metacognition; predictive frameworks situate both within a hierarchical inferential architecture. Rather than positing a single canonical deficit, a pluralist integration treats these approaches as complementary and selectively applicable to distinct subtypes or stages of hallucinatory experience.

A further axis along which a joint philosophical–scientific contribution is especially needed is *insight*, that is, the degree to which subjects recognize an experience as non-veridical. Philosophical treatments typically center on the indistinguishability of hallucinations from veridical perception (see §3.1); this can be read as targeting the limit case of lack of insight, characteristic of many psychotic hallucinations in which the non-veridical nature of the experience is not acknowledged. Such cases may indeed be regarded as actual occurrences of the perfect ‘philosophical hallucinations’, that is, experiences subjectively indistinguishable from veridical perception and lacking any external correlate. They represent limiting instances within the broader and heterogeneous spectrum of hallucinatory experience. On a *continuum* view, insight ranges from absent, through partial—a fluctuating acknowledgment of non-veridicality, as in some psychedelic-induced experiences and certain hypnagogic states—to preserved, common in Charles Bonnet syndrome, many hypnagogic and hypnopompic hallucinations, and frequently (though not uniformly) in psychedelic states. Importantly, lack of insight also occurs outside psychosis—for example, in disorders of body image such as the body dysmorphic disorder as well as in anorexia nervosa—raising questions about whether its source lies at the experiential/affective or the cognitive level and, more broadly, whether the affective–cognitive dichotomy is even adequate to explain the phenomena (Dolcini, 2024b). Framed this way, the notion of ‘philosophical’ hallucination is not orthogonal to clinical reality but isolates a dimension that structures empirical phenomena. Operationally, insight can be linked to source-monitoring performance, confidence, attribution judgments, and metacognitive indices, and it can be tracked alongside measures of agency, ownership, and sense of reality. Incorporating insight explicitly helps align conceptual analysis with clinical taxonomy and clarifies why apparently similar contents can play different epistemic and therapeutic roles across conditions.

The foregoing analysis shows both the richness and the conceptual instability of current approaches to hallucination. Three implications follow. First, hallucinations can be hardly conceived as a unitary category. Empirical evidence shows they differ across sensory modalities, clinical contexts, and neural mechanisms. Broad diagnostic categories risk neglecting fine-grained phenomenological differences that matter for conceptual analysis. Recognizing pluralism is crucial: hallucinations are better seen as a family of phenomena rather than a single kind. A second implication concerns the relation between cognition and perception. Bottom-up, top-down, and predictive processing models locate the ‘abnormality’ in different places, yet the traditional dichotomy between perceptual and cognitive explanations may obscure more than it clarifies. Predictive accounts suggest that hallucinations emerge from the same inferential mechanisms that underlie ordinary perception, differing only in the weighting of priors and prediction errors. This continuity raises questions about whether hallucinations are best framed as misperceptions, disordered cognitions, or phenomena that require a revision of this taxonomy.

Third, a genuinely interdisciplinary approach may benefit from the contribution of diverse research methodologies. Philosophy has a distinctive role in clarifying categories (e.g., separating indistinguishability from insight, ownership from agency, and articulating ‘sense of reality’), thereby preventing equivocation and guiding empirical work toward more precise taxonomies. Empirical research, in turn, constrains and enriches philosophical theorizing by revealing heterogeneity and boundary cases that test conceptual proposals. Interdisciplinary perspectives informed by phenomenology and clinical psychology already partially exemplify this alignment, but further work is needed to consolidate shared frameworks and to operationalize dimensions—such as insight, agency, sense of reality, and ownership—across methods and models.

## 5 Concluding remark

Hallucinations stand at the crossroads of philosophy, psychology, and neuroscience, and their conceptual analysis demands sensitivity to this plurality. The evidence reviewed here suggests that no single explanatory framework can account for all forms of hallucinatory experience. What emerges instead is a case for pluralistic taxonomies that respect differences across modalities, contexts, and mechanisms, and for integrative models that place existing accounts in complementary relation. By aligning their resources, philosophy and science can move toward a more productive dialogue, enriching both theoretical accounts and practical understanding of anomalous experience. Importantly, philosophy contributes here not only through interdisciplinary engagement with clinical or cognitive science, but also *via* conceptual analysis in the strict sense. The stipulative notion of hallucination as a subjectively indistinguishable experience, while abstract, remains illuminating: it sharpens the categories within which empirical findings are interpreted and maps onto the dimension of insight that structures many clinical cases. In this way, pure philosophical work and empirical research are not opposed but complementary, each clarifying aspects of a shared and heterogeneous phenomenon.

## Footnotes

1 Wilkinson et al. (2022) argue that ‘philosophy matters’ to the science of hallucinations. However, while their account valuably showcases philosophical work tied to actual cases, it gives less attention to the role of philosophy in articulating the conceptual core of hallucination, notably the indistinguishability condition.

2 For example, Macpherson and Batty (2016) argue that this traditional distinction is both conceptually and empirically inadequate, since many experiences (e.g., partial hallucinations, property illusions, and mixed cases), do not fit neatly into this binary scheme.

3 In neurodegenerative and ophthalmological conditions such as Parkinson’s disease, dementia with Lewy bodies, and Charles Bonnet syndrome, patients often exhibit not only hallucinations but also other visual anomalies, which may share partially overlapping neural substrates and prognostic implications (ffytche, 2007a, 2013, 2020).

4 In this view, post-lesion hallucinations are regarded as typically modality-specific and often recognized by patients as non-veridical, distinguishing them from the release of dream-like activities once posited by Lhermitte (1922).

5 For instance, recurrent complex visual hallucinations, like those reported in dementia with Lewy bodies or in Parkinson’s disease (Mosimann et al., 2006), often exhibit rich, structured content that appears to depend, at least partially, on higher-level constraints.

6 Hoffman based the model on previous observations suggesting a link between AVHs and speech disorganization (Andreasen et al., 1985; Hoffman et al., 1986). However, these results were widely dismissed as artifact of his selection process (Bentall and Slade, 1985), with later studies founding no reliable association between AVHs and speech incoherence (Liddle, 1987).

7 Predictive processing is not the only framework integrating top-down and bottom-up factors. In the study of visual hallucinations, for instance, several contemporary models combine both types of influence (Collerton et al., 2023), understanding complex visual hallucinations as emerging from dynamic interactions between sensory (bottom-up) and higher-order (top-down) processes.

8 While common kind theories and representationalism often overlap, they are not identical. Common kind theory stresses the sameness of the mental states involved in perception, illusion, and hallucination, whereas representationalism holds that perception essentially involves representing the world. One can still endorse common kind theory while rejecting representationalism (Johnston, 2004).

9 In principle, the positions can cross-cut: one may be a disjunctivist and still embrace representationalism (Tye, 2007), or a naïve realist while rejecting disjunctivism (Ali, 2018). Nevertheless, the simplification is not misleading: most relationalists are disjunctivists, and most representationalists reject both disjunctivism and relationalism.

10 It may be also observed that, as an anonymous reviewer insightfully noted, this tension is due, at least in part, to a methodological gap between scientific/clinical and philosophical inquiry—the former being predominantly probabilistic and statistical, the latter more logical and analytical. Yet philosophical methodology is far from homogeneous. Furthermore, developments such as Bayesian epistemology and experimental philosophy demonstrate that probabilistic frameworks can also ground philosophical analysis. One difference, however, may be individuated in the kinds of data each discipline relies on: while science primarily draws on empirical data (observable, measurable), philosophy may also engage with intuitions, phenomenological data, and linguistic data. In this sense, the evidential set available to philosophy is, in principle and potentially, broader than that of the empirical sciences (Dolcini, 2024a).

11 This serves his broader theory of apparent mental causation, which holds that the experience of will depends on three conditions: temporal priority of thought over action, consistency with prior thoughts, and the absence of alternative causes.
